# Hepatocellular Carcinoma Within the Milan Criteria: A Novel Inflammation-Based Nomogram System to Assess the Outcomes of Ablation

**DOI:** 10.3389/fonc.2020.01764

**Published:** 2020-09-14

**Authors:** Shuanggang Chen, Weimei Ma, Fei Cao, Lujun Shen, Han Qi, Lin Xie, Ying Wu, Weijun Fan

**Affiliations:** ^1^Department of Minimally Invasive Interventional Therapy, Sun Yat-sen University Cancer Center, Guangzhou, China; ^2^State Key Laboratory of Oncology in South China, Collaborative Innovation Center of Cancer Medicine, Sun Yat-sen University, Guangzhou, China; ^3^Department of Imaging Medicine, Sun Yat-sen University Cancer Center, Guangzhou, China

**Keywords:** hepatocellular carcinoma, nomogram, ablation techniques, inflammatory biomarkers, the Milan Criteria

## Abstract

**Objectives:**

Few studies based on pretreatment inflammation-based scores focused on assessing the prognosis of hepatocellular carcinoma (HCC) patients within the Milan Criteria after ablation. This study aimed to construct a nomogram based on a novel inflammation-based score for those patients.

**Methods:**

A total of 635 HCC patients within the Milan Criteria after ablation meeting the inclusion and exclusion criteria were included in the study. The novel inflammation-based score—Albumin-Platelet Score (APS)—was constructed by Cox proportional-hazards modeling. The nomogram based on APS was constructed by multivariate analysis and the “rms” R package. The performance of the APS and the nomogram were assessed by time-dependent receiver operating characteristic and the concordance index (C-index).

**Results:**

The APS was an integrated indicator based on peripheral albumin level and platelet counts, which was significantly superior to other inflammation-based scores (neutrophil to lymphocyte ratio, platelet to lymphocyte ratio, Prognostic Nutritional Index, modified Glasgow Prognostic Score, Glasgow Prognostic Score, Prognostic Index, and C-reactive protein/albumin ratio) in predicting the long-term prognosis of those patients undergoing ablation (*P* < 0.05). An easy-to-use nomogram based on three pretreatment clinical variables (i.e., the APS, tumor size, and age) was constructed and further improved significantly the performance in predicting the prognosis in patients within the Milan Criteria after ablation (*P* < 0.05). The C-index of nomogram for overall survival was 0.72 (95% CI 0.66, 0.77). The calibration plots with 1000 cycles of bootstrapping were well matched with the idealized 45° line.

**Conclusion:**

The APS was a better inflammation-based prognostic system than others. Also, the nomogram based on the APS improved the performance of predicting the prognosis of HCC patients within the Milan Criteria after ablation.

## KEY POINTS

• The Albumin-Platelet Score (APS) consisted of peripheral platelet counts and albumin level.
• The APS was superior to other inflammation-based scores in the performance of predicting the prognosis of hepatocellular carcinoma (HCC) patients within the Milan Criteria after ablation.
• The nomogram based on the APS improved the performance of predicting the prognosis of HCC patients within the Milan Criteria after ablation.

## Introduction

Hepatocellular carcinoma (HCC) accounts for 70–90% of liver cancer that was the fourth cancer-related death cause worldwide in 2018 (1). At present, the mainstream treatment of HCC within Milan Criteria (one lesion ≤5 cm or three lesions ≤3 cm without vascular invasion or extrahepatic metastasis) is still liver transplantation and surgical resection (2). However, with its advantages of minimal invasiveness and cost-effectiveness, local ablation treatment is recommended by the National Comprehensive Cancer Network as an optional first-line curative therapy for early HCC (3, 4).

Inflammation is considered as a hallmark of cancer, and more and more evidence has shown that inflammation is closely related to the progression, recurrence, and survival of patients with HCC (5, 6). Recently, different inflammation-based scores, such as the Glasgow Prognostic Score (GPS), modified Glasgow Prognostic Score (mGPS), Prognostic Index (PI), Prognostic Nutritional Index (PNI), neutrophil to lymphocyte ratio (NLR), platelet to lymphocyte ratio (PLR), and C-reactive protein/albumin ratio (CAR), have been proposed and been also thought to predict the prognosis of HCC, which mainly calculate quantitative values of plasma neutrophil count, total lymphocyte count, platelet count, albumin level and C-reactive protein (CRP) level, or the ratio or combination between the two indicators; however, those inflammation-based scores are not adequate to predict the overall survival (OS) of HCC patients (7–13). Besides, to our knowledge, the vast majority of studies on these pretreatment inflammation-based markers have not targeted patients with HCC within the Milan Criteria for ablation therapy. Therefore, we systematically analyzed the pre-treatment clinical characteristics and the inflammatory indicators included in these inflammation-based scores of patients with HCC within the Milan Criteria of ablation therapy and integrate a novel combination of inflammatory indicators—APS. Also, we hypothesized that the nomogram based on the APS could improve the performance of predicting the prognosis. To test this hypothesis, we constructed a simple and clinically applicable nomogram based on the APS to assess the prognosis of HCC patients within the Milan Criteria after curative ablation.

## Materials and Methods

**FIGURE 1:**
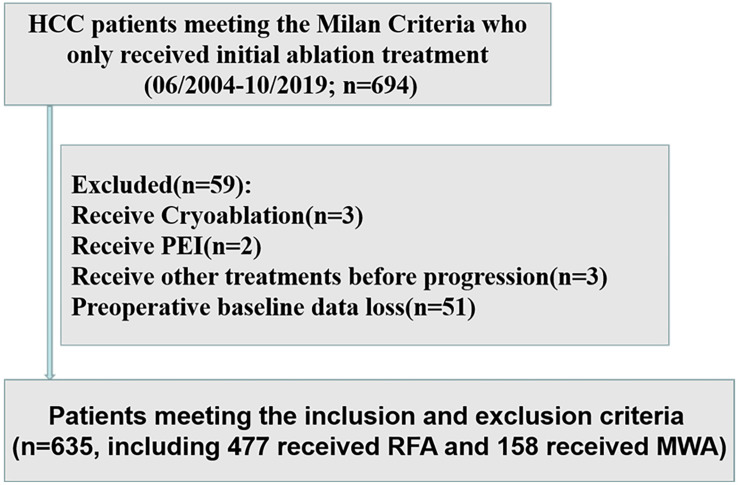
Flow diagram of the study design. HCC, hepatocellular carcinoma; PEI, percutaneous ethanol injection; MWA, microwave ablation; RFA, radiofrequency ablation.

We retrospectively analyzed the data of 694 HCC patients within the Milan Criteria at the Sun Yat-sen University Cancer Center (SYSUCC) between June 2004 and October 2019. The inclusion criteria included (1) patients with HCC diagnosis confirmed by radiologic imaging studies or histopathological examination, (2) HCC treated with initial radiofrequency ablation (RFA) or microwave ablation (MWA), and (3) HCC treated with curative ablation. The exclusion criteria were (1) severe coagulation disorders and renal dysfunction, (2) patients who receive cryoablation or percutaneous ethanol injection (PEI), (3) patients who receive other treatments for HCC except ablation before progression, and (4) patients with preoperative baseline data loss (Figure 1). After the application of these inclusion and exclusion criteria, a total of 635 HCC patients within the Milan Criteria were included in the study. The study was conducted in accordance with the Declaration of Helsinki and was approved by the SYSUCC Hospital Ethics Committee.

### Baseline Data Collection and Inflammation-Based Prognostic Scores

We collected the baseline data of those patients before initial ablation, including patient characteristics, imaging, biochemistry, tumor markers, coagulation, and blood routine. Important clinical data included patient characteristics (gender, age, HBV infection, treatment), imaging (tumor size, tumor numbers, cirrhosis), biochemistry [albumin (ALB), CRP, total bilirubin (TBIL), alanine aminotransferase (ALT), aspartate transaminase (AST)], tumor markers (alpha-fetoprotein), and coagulation [prothrombin time (PT)] blood routine [white blood cell (WBC), neutrophil, lymphocyte, monocyte, platelet]. The albumin-bilirubin (ALBI) score was defined as −0.085 × (albumin g/L) + 0.66 × log (TBIL μmol/L) (14). The APS, mGPS, GPS, PNI, PI, PLR, NLR, and CAR were constructed as described in Table 1.

**TABLE 1:** Systemic inflammation-based prognostic scores.

Scoring systems	Score
GPS	
CRP (≤10 mg/L) and albumin (≥35 g/L)	0
CRP (≤10 mg/L) and albumin (<35 g/L)	1
CRP (>10 mg/L) and albumin (≥35 g/L)	1
CRP (>10 mg/L) and albumin (<35 g/L)	2
mGPS	
CRP (≤10 mg/L)	0
CRP (>10 mg/L) and albumin (≥35 g/L)	1
CRP (>10 mg/L) and albumin (<35 g/L)	2
PI	
CRP (≤10 mg/L) and WBC (≤10 × 10^9^/L)	0
CRP (≤10 mg/L) and WBC (>10 × 10^9^/L)	1
CRP (>10 mg/L) and WBC (≤10 × 10^9^/L)	1
CRP (>10 mg/L) and WBC (>10 × 10^9^/L)	2
PNI	
Albumin (g/L) + 5 × total lymphocyte count (×10^9^/L) ≥ 45	0
Albumin (g/L) + 5 × total lymphocyte count (×10^9^/L) < 45	1
NLR	
Neutrophil count (×10^9^/L): lymphocyte count (×10^9^/L) < 3	0
Neutrophil count (×10^9^/L): lymphocyte count (×10^9^/L) ≥ 3	1
PLR	
Platelet count (× 10^9^/L): lymphocyte count (×10^9^/L) < 150	0
Platelet count (× 10^9^/L): lymphocyte count (×10^9^/L) ≥ 150	1
CAR	
CRP (mg/L): albumin (g/L) < 0.05	0
0.05 ≤ CRP (mg/L): albumin (g/L) < 0.1	1
CRP (mg/L): albumin (g/L) ≥ 0.1	2
APS	
Albumin > 37.7 g/L, PLT > 80 × 10^9^/L	1
Albumin > 37.7 g/L, PLT ≤ 80 × 10^9^/L	2
Albumin ≤ 37.7 g/L, PLT > 80 × 10^9^/L	2
Albumin ≤ 37.7 g/L, PLT ≤ 80 × 10^9^/L	3

*WBC, white blood cell; PLT, platelet; CRP, C-reactive protein; GPS, Glasgow Prognostic Score; NLR, neutrophil to lymphocyte ratio; mGPS, modified Glasgow Prognostic Score; PI, Prognostic Index; PLR, platelet to lymphocyte ratio; PNI, Prognostic Nutritional Index; CAR, C-reactive protein/albumin ratio; APS, Albumin-Platelet Score.*

### Treatment Protocols

Microwave ablation and radiofrequency ablation procedures were performed under real-time ultrasound (US) or CT by radiologists who had at least 5 years of experience in interventional therapy. Both therapies were administered after analgesia (50–60 mg propofol and 0.05–0.1 mg fentanyl) and local anesthesia (5–15 mL 1–2% lidocaine) by anesthesiologists. According to the location, size, and number of the lesions, radiologists chose the number of ablation antennas, the power and corresponding time and whether to adjust the needle position in order to eliminate tumors. The basic principles of ablation treatment are as follows. For tumors with a maximum diameter of ≤3.0 cm, a single antenna was usually used. For tumors with a maximum diameter of >3.0 cm, multiple antennas were usually used to acquire adequate ablation necrosis. The end point of ablation was defined as having a security boundary that extended at least 5–10 mm beyond the tumor boundary.

Microwave ablation equipment: a microwave delivery system (FORSEA; Qinghai Microwave Electronic Institute, Nanjing, China) was used during MWA therapy. This system consisted of an MTC-3 microwave generator (FORSEA) with a frequency of 2450 MHz, a power output of 10–150 W, a flexible low-loss cable, and a 15- or 18-cm 14G or 16G cooled-shaft antenna.

Radiofrequency ablation equipment: radiofrequency system (RF 2000; RadioTherapeutics, Mountain View, CA, United States) and a needle electrode with a 15G insulated cannula with 10 hook-shaped expandable electrode tines with a diameter of 3.5 cm at expansion (LeVeen; RadioTherapeutics).

### Following Up

Follow-up included the imaging examination, serum AFP, the liver function, and the physical examination. Patients underwent a re-examination approximately 1 month after RFA or MWA treatment using abdominal contrast material-enhanced CT, US, or MRI. If there were no obvious signs of recurrence, those patients were followed up once every 3 months for the first 2 years. If recurrence was still not observed, the follow-up visits were allowed to extend to once every 6 months from 2 to 5 years after RFA or MWA and then to once every 12 months after 5 years. If recurrence was detected, the patients were allowed to treat with RFA or MWA, transarterial chemoembolization (TACE), systemic chemotherapy, targeted therapy, or supportive treatment according to the patient’s physical condition, liver function, and the tumor staging at the time of tumor recurrence. Technical success was defined as the diameter of the non-enhanced area being greater than that of the treated nodule. The end point, OS, was defined as the interval time from the start of initial RFA or MWA treatment to death by any cause.

### Statistical Analysis

Continuous variables that met the normal distribution were described by mean ± SD, otherwise by median and quartile. Continuous variables were compared by using the *t*-test or Mann–Whitney U test. Binary variables were compared by using the χ^2^ test or the Fisher exact test. Also, ordinal categorical variables were compared by using the Kruskal–Wallis H test. The optimal cut-off value of baseline variables was calculated by “survivalROC” R package (15). Those baseline variables were included in a time-dependent Cox proportional-hazards modeling for univariate analysis. Variables satisfying *P* < 0.1 in univariate analysis were introduced into the multivariate time-dependent Cox proportional-hazards modeling. The OS rate between the different groups were compared by Kaplan–Meier curves and log-rank test. The abilities to predict prognosis of the variables with respect to OS were compared by time-dependent receiver operating characteristic (ROC) curves and the estimated area under the curve (AUC). The concordance index (C-index) and time-dependent ROC were analyzed by using the “survival” and “timeROC” R package (16). A nomogram was constructed based on the results of multivariate time-dependent Cox proportional-hazards modeling and by the “rms” R package. The C-index, the internal validation with 1000 sets of bootstrap samples, and the calibration curve were used to demonstrate ability to predict prognosis of the nomogram model. Analyses were two-sided, and *P* < 0.05 indicated statistical significance. Statistical analyses were conducted using SPSS version 25.0 (IBM, United States) and R version 3.6.1^1^.

## Results

### Patient Characteristics

A total of 635 HCC patients within the Milan Criteria meeting the inclusion and exclusion criteria were included in this study. The mean age of those patients was 57.74 years (57.74 ± 12.35 years). The median size was 2.30 cm (range: 0.70–5.00 cm). A total of 577 (90.9%) and 58 (9.1%) of HCC patients had solitary and multiple tumors, respectively. There were 573 (90.2%) patients with hepatitis B virus (HBV) infection and 353 (55.6%) patients with cirrhosis, respectively. A total of 477 (75.1%) and 158 (24.9%) of HCC patients were treated with RFA and MWA, respectively. Other clinical characteristics and the inflammation-based scores are depicted in Table 2.

**TABLE 2:** Demographic and clinical characteristics of the enrolled patients.

Variables	*N* = 635 or median (*n*% or interquartile Q_1_–Q_3_)
Gender (male vs. female)	531 vs. 104 (83.6 vs. 16.4)
Age (years)	57.74 ± 12.35
ALB (g/L)	42.10 (39.00, 45.10)
Cirrhosis (absent vs. present)	282 vs. 353 (44.4 vs. 55.6)
HBV infection (absent vs. present)	62 vs. 573 (9.8 vs. 90.2)
TBIL (μmol/L)	14.30 (10.90, 20.20)
WBC (×10^9^/L)	5.26 (4.18, 6.50)
Neutrophil count (×10^9^/L)	2.80 (2.10, 3.75)
Lymphocyte count (×10^9^/L)	1.60 (1.20, 2.06)
Monocyte count (×10^9^/L)	0.40 (0.30, 0.50)
Prothrombin time (s)	12.20 (11.50, 13.10)
PLT (×10^9^/L)	131.00 (87.00, 177.00)
CRP (mg/L)	1.25 (0.66, 2.59)
ALT (U/L)	32.00 (22.10, 47.90)
AST (U/L)	32.40 (25.00, 44.60)
AFP (<37.15 ng/ml vs. ≥ 37.15 ng/ml)	328 vs. 307 (51.7 vs. 48.3)
Tumor size (<3.5 cm vs. ≥3.5 cm)	573 vs. 62 (90.2 vs. 9.8)
Tumor numbers (solitary vs. multiple)	577 vs. 58 (90.9 vs. 9.1)
Treatment (RFA vs. MWA)	477 vs. 158 (75.1 vs. 24.9)
ALBI grade (1 vs. 2 vs. 3)	438 vs. 194 vs. 3 (69.0 vs. 30.5 vs. 0.5)
GPS before treatment (0/1/2)	543 vs. 84 vs. 8 (85.5 vs. 13.2 vs. 1.3)
NLR before treatment (0/1)	530 vs. 105 (83.5 vs. 16.5)
mGPS before treatment (0/1/2)	601 vs. 26 vs. 8 (94.6 vs. 4.1 vs. 1.3)
PI before treatment (0/1/2)	596 vs. 31 vs. 8 (93.9 vs. 4.9 vs. 1.2)
PLR before treatment (0/1)	586 vs. 49 (92.3 vs. 7.7)
PNI before treatment (0/1)	502 vs. 133 (79.1 vs. 20.9)
CAR before treatment (0/1/2)	429 vs. 100 vs. 106 (67.6 vs. 15.7 vs. 16.7)

*ALB, albumin; HBV, hepatitis B virus; TBIL, total bilirubin; WBC, white blood cell; PLT, platelet; CRP, C-reactive protein; ALT, alanine aminotransferase; AST, aspartate transaminase; ALBI, albumin-bilirubin; GPS, Glasgow Prognostic Score; NLR, neutrophil to lymphocyte ratio; mGPS, modified Glasgow Prognostic Score; PI, Prognostic Index; PLR, platelet to lymphocyte ratio; PNI, Prognostic Nutritional Index.*

### Optimal Cut-Off Value of Baseline Variables

The optimal cut-off value of baseline variables was calculated by survival ROC, which could fit Cox proportional-hazards modeling to the status and the time of survival. The optimal cut-off value of tumor size, AFP level, PT, ALB, TBIL, WBC, neutrophil, lymphocyte, monocyte, platelet, C-reactive protein (CRP), ALT, and AST were 3.5 cm, 37.15 ng/ml, 13.6 s, 37.7 g/L, 28.3 μmol/L, 4.24 × 10^9^/L, 2.41 × 10^9^/L, 1.43 × 10^9^/L, 0.64 × 10^9^/L, 80 × 10^9^/L, 1.81 mg/L, 52.5 U/L, and 41.0 U/L, respectively.

### Establishment of the Inflammation-Based Score—APS

**FIGURE 2:**
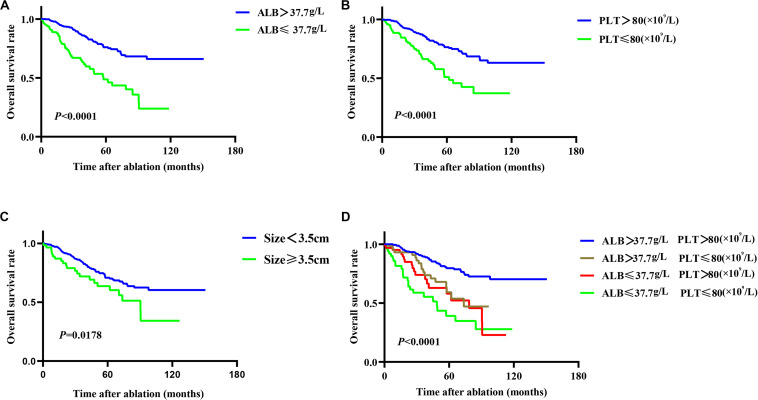
Kaplan–Meier plots for independent prognostic factors of overall survival (OS) in patients with HCC within the Milan Criteria after RFA. **(A,B)** Patients with reduced albumin (ALB) and platelet (PLT) level had lower OS rate than did those with higher ALB and PLT level. **(C)** Patients with tumor size ≥3.5 cm had lower OS rate than did those with size <3.5 cm. **(D)** Different combinations of ALB and PLT showed the different median OS, but the OS of the two combinations of (ALB > 37.7 g/L, PLT ≤ 80 × 10^9^/L) and (ALB ≤ 37.7 g/L, PLT > 80 × 10^9^/L) did not reach statistical difference (*P* = 0.397).

Twenty-seven variables (gender, tumor size, tumor numbers, AFP level, HBV infection, treatment method, cirrhosis, PT, ALB, TBIL, WBC, neutrophil, lymphocyte, monocyte, PLT, CRP, ALT, AST, age, ALBI grade, NLR, PLR, PNI, mGPS, GPS, PI, CAR) were included in the time-dependent Cox proportional-hazards modeling one by one for univariate analysis, and we introduced those variables satisfying *P* < 0.1 in univariate analysis into the multivariate time-dependent Cox proportional-hazards modeling, and found that only four variables (tumor size, ALB, PLT, age) were independent prognostic factors of OS (Table 3 and Figure 2A–C). Therefore, we combined ALB with PLT (i.e., ALB + PLT) to construct a novel inflammation-based prognostic score. The OS rate between the different groups of ALB + PLT was compared by Kaplan–Meier curves and log-rank test (Figure 2D). As shown in Figure 2D, we combined (ALB > 37.7 g/L, PLT ≤ 80 × 10^9^/L) and (ALB ≤ 37.7 g/L, PLT > 80 × 10^9^/L) of ALB + PLT and recorded it as APS 2 level. We then included those variables satisfying *P* < 0.1 in univariate analysis and APS into proportional-hazards modeling for multivariate analysis, and found that only three variables (tumor size, APS, age) were independent prognostic factors for the OS of HCC patients within the Milan Criteria after ablation (Table 4).

**TABLE 3:** Univariate and multivariate of the prognostic factors for overall survival based on time-dependent Cox regression analyses.

Variable	Number of cases	Univariate analysis		Multivariate analysis	
		HR (95% CI)	*P-*value	HR (95% CI)	*P-*value
Gender (female vs. male)	104 vs. 531	1.44 (0.90–2.31)	0.125	–	–
Tumor size (≥3.5 cm vs. <3.5 cm)	62 vs. 573	1.75 (1.10–2.80)	0.019	2.09 (1.29–3.37)	0.003
AFP level (≥37.15 ng/ml vs. <37.15 ng/ml)	307 vs. 328	1.44 (0.98–2.10)	0.060	–	0.162
HBV infection (present vs. absent)	573 vs. 62	1.68 (0.78–3.61)	0.185	–	–
Numbers (multiple vs. solitary)	58 vs. 577	1.29 (0.70–2.33)	0.423	–	–
Treatment (MWA vs. RFA)	158 vs. 477	1.02 (0.64–1.61)	0.949	–	–
Cirrhosis (present vs. absent)	353 vs. 282	1.59 (1.08–2.36)	0.019	–	0.994
PT (s) (≥13.6 vs. <13.6)	104 vs. 531	2.69 (1.80–4.01)	<0.001	–	0.100
ALB (≤37.7 g/L vs. >37.7 g/L)	120 vs. 515	3.20 (2.19–4.68)	<0.001	2.76 (1.84–4.16)	<0.001
TBIL (≥28.3 μmol/L vs. <28.3 μmol/L)	66 vs. 569	2.22 (1.38–3.58)	0.001	–	0.359
WBC (≤4.24 × 10^9^/L vs. >4.24 × 10^9^/L)	175 vs. 460	1.71 (1.16–2.52)	0.007	–	0.955
Neutrophil (≤2.41 × 10^9^/L vs. >2.41 × 10^9^/L)	231 vs. 404	1.65 (1.14–2.40)	0.009	–	0.437
Lymphocyte (≤1.43 × 10^9^/L vs. >1.43 × 10^9^/L)	250 vs. 385	1.54 (1.06–2.23)	0.025	–	0.867
Monocyte (≥0.64 × 10^9^/L vs. <0.64 × 10^9^/L)	57 vs. 578	1.37 (0.80–2.32)	0.250	–	–
PLT (≤80 × 10^9^/L vs. >80 × 10^9^/L)	134 vs. 501	2.57 (1.75–3.77)	<0.001	2.04 (1.36–3.05)	0.001
CRP (≥1.81 mg/L vs. <1.81 mg/L)	230 vs. 405	1.82 (1.26–2.65)	0.002	–	0.413
ALT (≥52.5 U/L vs. <52.5 U/L)	127 vs. 508	1.00 (0.63–1.59)	0.998	–	–
AST (≥41.0 U/L vs. <41.0 U/L)	200 vs. 435	2.15 (1.48–3.12)	<0.001	–	0.132
Age (years)	635	1.03 (1.01–1.04)	0.001	1.03 (1.01–1.05)	<0.001
ALBI grade before treatment			<0.001		0.316
1	438	Reference		Reference	
2	194	2.91 (1.99–4.25)	<0.001	–	0.196
3	3	3.03 (0.42–22.02)	0.273	–	0.623
NLR before treatment (1 vs. 0)	105 vs. 530	1.23 (0.77–1.99)	0.387	–	–
PLR before treatment (1 vs. 0)	49 vs. 586	0.95 (0.42–2.17)	0.908	–	–
PNI before treatment (1 vs. 0)	133 vs. 502	2.39 (1.63–3.53)	<0.001	–	0.535
mGPS before treatment			0.084		0.946
0	601	Reference		Reference	
1	26	1.31 (0.53–3.21)	0.557	–	0.897
2	8	3.03 (1.12–8.25)	0.030	–	0.753
GPS before treatment			<0.001		0.405
0	543	Reference		Reference	–
1	84	2.95 (1.93–4.53)	<0.001	–	0.179
2	8	3.67 (1.34–10.05)	0.011	–	0.753
PI before treatment			0.128		–
0	596	Reference		–	–
1	31	1.96 (0.99–3.87)	0.054	–	–
2	8	0.56 (0.08–4.01)	0.563	–	–
CAR before treatment			0.004		0.845
0	429	Reference	–	Reference	–
1	100	1.67 (1.03–2.71)	0.037	–	0.758
2	106	2.03 (1.30–3.18)	0.002	–	0.566

*ALB, albumin; HBV, hepatitis B virus; TBIL, total bilirubin; WBC, white blood cell; PLT, platelet; CRP, C-reactive protein; ALT, alanine aminotransferase; AST, aspartate transaminase; ALBI, albumin-bilirubin; GPS, Glasgow Prognostic Score; NLR, neutrophil to lymphocyte ratio; mGPS, modified Glasgow Prognostic Score; PI, Prognostic Index; PLR, platelet to lymphocyte ratio; PNI, Prognostic Nutritional Index.*

**TABLE 4:** Multivariate of the prognostic factors for overall survival based on time-dependent Cox regression analyses.

Variable	Number of cases	Multivariate analysis	
		HR (95% CI)	*P-*value
Cirrhosis (present vs. absent)	353 vs. 282	–	0.954
PT (s) (≥13.6 vs. <13.6)	104 vs. 531	–	0.088
TBIL (≥28.3 μmol/L vs. <28.3 μmol/L)	66 vs. 569	–	0.335
WBC (≤4.24 × 10^9^/L vs. >4.24 × 10^9^/L)	175 vs. 460	–	0.819
Lymphocyte (≤1.43 × 10^9^/L vs. >1.43 × 10^9^/L)	250 vs. 385	–	0.687
CRP (≥1.81 mg/L vs. <1.81 mg/L)	230 vs. 405	–	0.316
AST (≥41.0 U/L vs. <41.0 U/L)	200 vs. 435	–	0.119
ALB (≤37.7 g/L vs. >37.7 g/L)	120 vs. 515	–	0.357
PLT (≤80 × 10^9^/L vs. >80 × 10^9^/L)	134 vs. 501	–	0.357
Neutrophil (≤2.41 × 10^9^/L vs. >2.41 × 10^9^/L)	231 vs. 404	–	0.514
PNI before treatment (1 vs. 0)	133 vs. 502	–	0.725
AFP level (≥37.15 ng/ml vs. <37.15 ng/ml)	307 vs. 328	–	0.141
Size (≥3.5 cm vs. <3.5 cm)	62 vs. 573	1.99 (1.24–3.22)	0.005
Age (years)	635	1.03 (1.01–1.05)	<0.001
mGPS before treatment			0.977
0	601	Reference	–
1	26	–	0.863
2	8	–	0.888
GPS before treatment			0.251
0	543	Reference	–
1	84	–	0.100
2	8	–	0.888
ALBI grade before treatment			0.230
1	438	Reference	
2	194	–	0.129
3	3	–	0.556
CAR before treatment			0.737
0	429	Reference	
1	100	–	0.806
2	106	–	0.435
APS before treatment			<0.001
1 grade	433	Reference	–
2 grade	150	2.52 (1.64–3.87)	<0.001
3 grade	52	5.51 (3.35–9.05)	<0.001

*ALB, albumin; HBV, hepatitis B virus; TBIL, total bilirubin; WBC, white blood cell; PLT, platelet; CRP, C-reactive protein; AST, aspartate transaminase; ALBI, albumin-bilirubin; GPS, Glasgow Prognostic Score; NLR, neutrophil to lymphocyte ratio; mGPS, modified Glasgow Prognostic Score; PNI, Prognostic Nutritional Index.*

### The Performance and Discrimination of the APS

**FIGURE 3:**
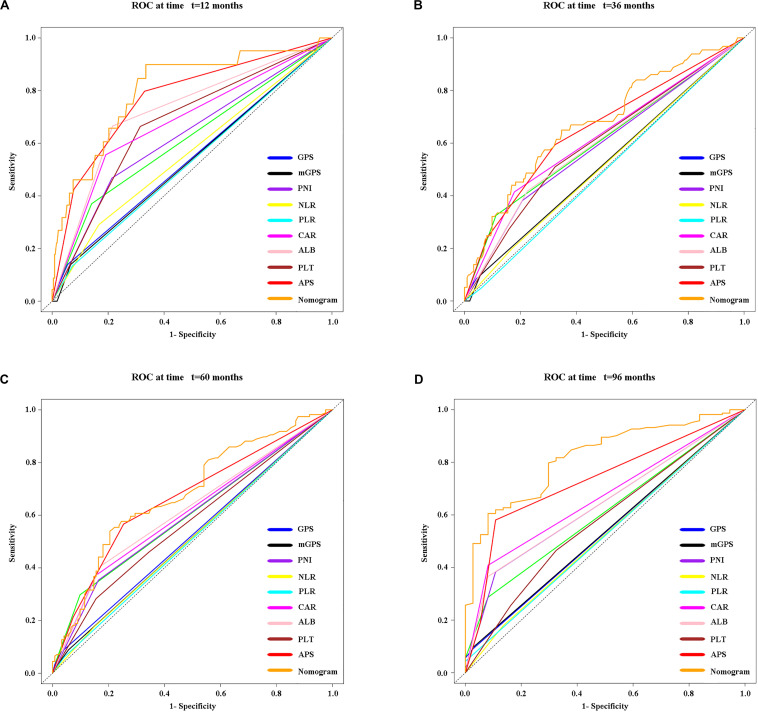
Time-dependent receiver operating characteristic (timeROC) curves at 1 **(A)**, 3 **(B)**, 5 **(C)**, and 8 **(D)** years of OS based on different inflammation-based scores, variables (i.e., ALB and PLT) that built the APS, and the nomogram based on the three pretreatment clinical variables, including the APS level, tumor size, and age.

**FIGURE 4:**
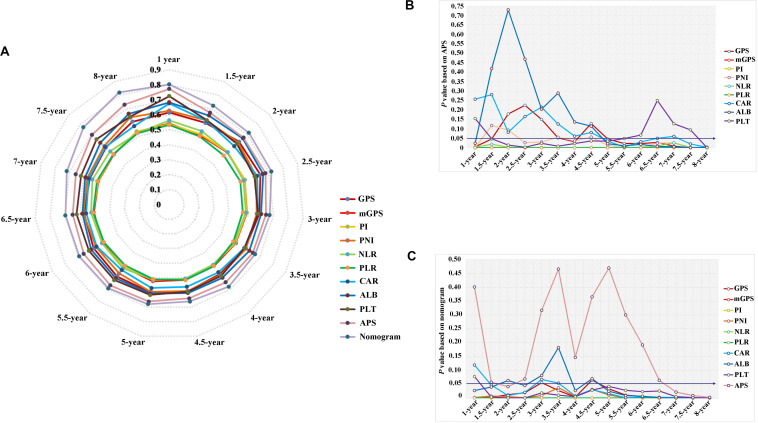
The comparison of serial trends of their performance and discrimination of different inflammation-based scores, variables that built the APS, and the nomogram by the estimated area under the curve (AUC) values **(A)**, and the corresponding *P-*value based on APS **(B)** and the nomogram **(C)**.

Time-dependent ROC curves at 1, 3, 5, and 8 years of OS were constructed to compare the performance of the other inflammation-based scores and variables (i.e., ALB and PLT) that built APS, which suggested that APS was superior to other factors (Figure 3). The details of the corresponding AUC values and C-index values of those variables for OS prediction are depicted in Table 5, which showed that the AUC values and C-index value (0.67; 95% CI 0.62, 0.73) of APS was higher than that of others. To further prove the performance and discrimination of APS, the AUC values (Figure 4A) and corresponding *P-*value based on APS (Figure 4B) of those inflammation-based scores, ALB, and PLT at different times were calculated to compare the sequential trends of their performance and discrimination, which showed that APS was significantly superior to other factors in predicting the long-term prognosis.

**TABLE 5:** Comparison of the performance and discriminative ability between the preoperative blood-related prognostic factors.

Score	1-year AUC (95% CI)	3-year AUC (95% CI)	5-year AUC (95% CI)	8-year AUC (95% CI)	C-index (95% CI)
ALB	0.68 (0.57, 0.79)	0.62 (0.55, 0.68)	0.61 (0.55, 0.67)	0.66 (0.59, 0.73)	0.64 (0.59, 0.69)
PLT	0.72 (0.62, 0.83)	0.60 (0.53, 0.66)	0.62 (0.56, 0.68)	0.64 (0.57, 0.71)	0.61 (0.56, 0.67)
PLR	0.53 (0.46, 0.60)	0.49 (0.46, 0.52)	0.51 (0.48, 0.54)	0.52 (0.50, 0.54)	0.50 (0.47, 0.53)
mGPS	0.54 (0.47, 0.62)	0.52 (0.49, 0.56)	0.52 (0.49, 0.56)	0.53 (0.49, 0.58)	0.52 (0.49, 0.55)
PI	0.54 (0.46, 0.61)	0.52 (0.48, 0.56)	0.52 (0.48, 0.55)	0.53 (0.49, 0.58)	0.52 (0.49, 0.55)
NLR	0.56 (0.46, 0.66)	0.51 (0.46, 0.57)	0.51 (0.46, 0.56)	0.52 (0.45, 0.59)	0.52 (0.48, 0.57)
PNI	0.63 (0.52, 0.74)	0.59 (0.52, 0.65)	0.59 (0.53, 0.66)	0.64 (0.56, 0.71)	0.60 (0.55, 0.65)
GPS	0.61 (0.51, 0.72)	0.61 (0.55, 0.67)	0.60 (0.54, 0.66)	0.61 (0.54, 0.67)	0.60 (0.55, 0.65)
CAR	0.68 (0.57, 0.78)	0.60 (0.52, 0.67)	0.57 (0.50, 0.64)	0.57 (0.48, 0.67)	0.60 (0.54, 0.65)
APS	0.77 (0.67, 0.88)	0.65 (0.58, 0.72)	0.66 (0.59, 0.73)	0.73 (0.65, 0.81)	0.67 (0.62, 0.73)
Nomogram	0.80 (0.70, 0.91)	0.67 (0.60, 0.75)	0.68 (0.61, 0.75)	0.82 (0.75, 0.89)	0.71 (0.66, 0.77)

### Correlations Between Patient Characteristics and the APS

**FIGURE 5:**
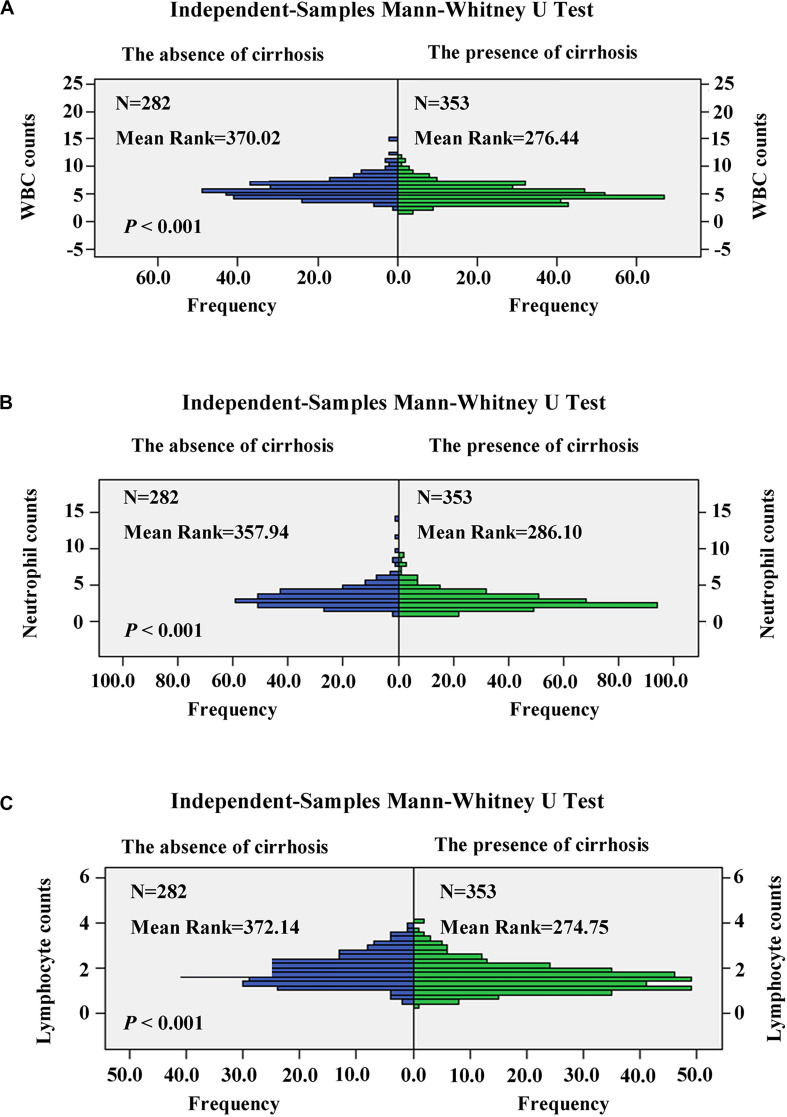
Comparison of white blood cell (WBC) **(A)**, neutrophil **(B)**, and lymphocyte **(C)** counts in patients with cirrhosis and non-cirrhosis by the independent-samples Mann–Whitney U test.

The relationship between the APS and patient characteristics is summarized in Table 6. The higher APS was significantly associated with female (*P* = 0.006); cirrhosis (*P* < 0.001); PT ≥ 13.6 s (*P* < 0.001); TBIL ≥ 28.3 μmol/L (*P* < 0.001); WBC ≤ 4.24 × 10^9^/L (*P* < 0.001); neutrophil ≤ 2.41 × 10^9^/L (*P* < 0.001); lymphocyte ≤ 1.43 × 10^9^/L (*P* < 0.001); CRP ≥ 1.81 mg/L (*P* < 0.001); AST ≥ 41.0 U/L (*P* < 0.001); older patients (*P* < 0.001); increased ALBI grade (*P* < 0.001); and increased PNI, mGPS, GPS, and CAR (*P* < 0.001). Besides, patients with cirrhosis have significantly reduced WBC (*P* < 0.001), neutrophil (*P* < 0.001), and lymphocyte (*P* < 0.001) counts than patients without cirrhosis (Figure 5).

**TABLE 6:** Clinical characteristics of patients in relation to APS.

Variables	APS 1 grade	APS 2 grade	APS 3 grade	*P-*value
	*N* = 433	*N* = 150	*N* = 52	
Gender (female vs. male)	57 vs. 376	35 vs. 115	12 vs. 40	0.006
Tumor size (≥3.5 cm vs. <3.5 cm)	39 vs. 394	18 vs. 132	5 vs. 47	0.567
AFP level (≥37.15 ng/ml vs. <37.15 ng/ml)	198 vs. 235	79 vs. 71	30 vs. 22	0.127
HBV infection (present vs. absent)	392 vs. 41	134 vs. 16	47 vs. 5	0.913
Numbers (multiple vs. solitary)	33 vs. 400	17 vs. 133	8 vs. 44	0.105
Treatment (MWA vs. RFA)	101 vs. 332	39 vs. 111	18 vs. 34	0.192
Cirrhosis (present vs. absent)	193 vs. 240	118 vs. 32	42 vs. 10	<0.001
PT(s) (≥13.6 vs. <13.6)	21 vs. 412	47 vs. 103	36 vs. 16	<0.001
TBIL (≥28.3 μmol/L vs. <28.3 μmol/L)	21 vs. 412	24 vs. 126	21 vs. 31	<0.001
WBC (≤4.24 × 10^9^/L vs. >4.24 × 10^9^/L)	69 vs. 364	72 vs. 78	34 vs. 18	<0.001
Neutrophil (≤2.41 × 10^9^/L vs. >2.41 × 10^9^/L)	111 vs. 322	83 vs. 67	37 vs. 15	<0.001
Lymphocyte (≤1.43 × 10^9^/L vs. >1.43 × 10^9^/L)	130 vs. 303	80 vs. 70	40 vs. 12	<0.001
Monocyte (≥0.64 × 10^9^/L vs. <0.64 × 10^9^/L)	39 vs. 394	14 vs. 136	4 vs. 48	0.938
CRP (≥1.81 mg/L vs. <1.81 mg/L)	129 vs. 304	68 vs. 82	33 vs. 19	<0.001
ALT (≥52.5 U/L vs. <52.5 U/L)	84 vs. 349	29 vs. 121	14 vs. 38	0.428
AST (≥41.0 U/L vs. <41.0 U/L)	95 vs. 338	69 vs. 81	36 vs. 16	<0.001
Age (years)	57 (48–66)*	60 (52–69)*	59 (51–66)*	0.039
ALBI grade before treatment				<0.001
1	385	53	0	–
2	48	95	51	–
3	0	2	1	–
NLR before treatment (1 vs. 0)	65 vs. 368	30 vs. 120	10 vs. 42	0.316
PLR before treatment (1 vs. 0)	40 vs. 393	7 vs. 143	2 vs. 50	0.108
PNI before treatment (1 vs. 0)	17 vs. 416	67 vs. 83	49 vs. 3	<0.001
mGPS before treatment				<0.001
0	416	141	44	–
1	17	6	3	–
2	0	3	5	–
GPS before treatment				<0.001
0	416	111	16	–
1	17	36	31	–
2	0	3	5	–
PI before treatment				0.064
0	411	141	44	–
1	18	7	6	–
2	4	2	2	–
CAR before treatment				<0.001
0	326	85	18	–
1	54	33	13	–
2	53	32	21	–

ALB, albumin; HBV, hepatitis B virus; TBIL, total bilirubin; WBC, white blood cell; PLT, platelet; CRP, C-reactive protein; ALT, alanine aminotransferase; AST, aspartate transaminase; ALBI, albumin-bilirubin; GPS, Glasgow Prognostic Score; NLR, neutrophil to lymphocyte ratio; mGPS, modified Glasgow Prognostic Score; PI, Prognostic Index; PLR, platelet to lymphocyte ratio; PNI, Prognostic Nutritional Index. *These data represent the median (interquartile Q1–Q3).

### Construction and Validation of Nomogram Based on the APS

**FIGURE 6:**
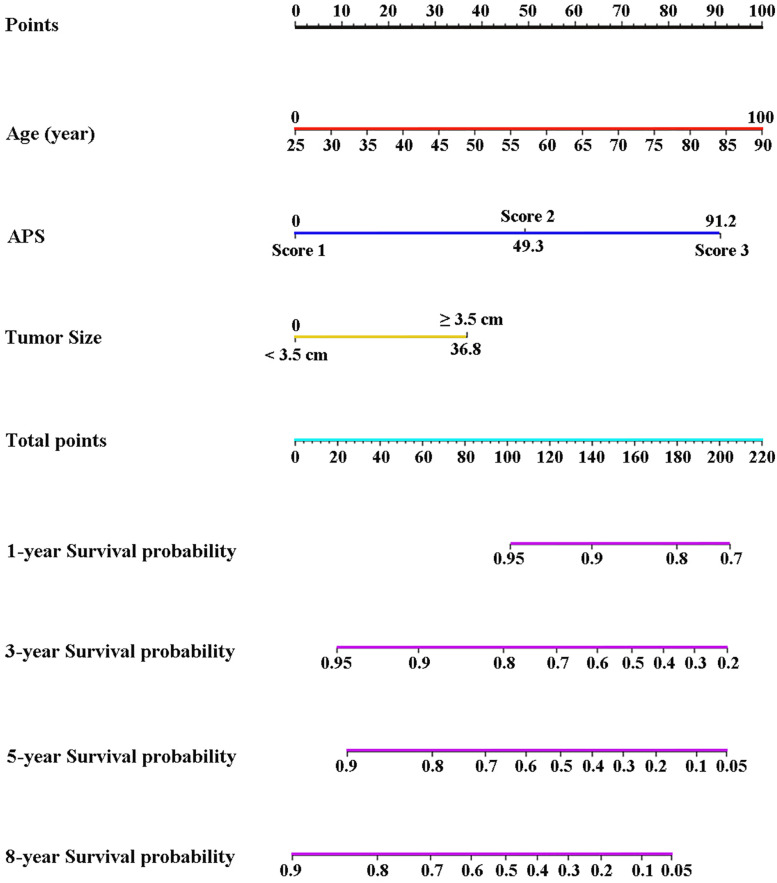
Nomogram based on the three pretreatment clinical variables, including APS level, tumor size, age, showed assessment of 1-, 3-, 5-, and 8-year OS of HCC patients within the Milan Criteria after ablation. APS, Albumin-Platelet Score.

**FIGURE 7:**
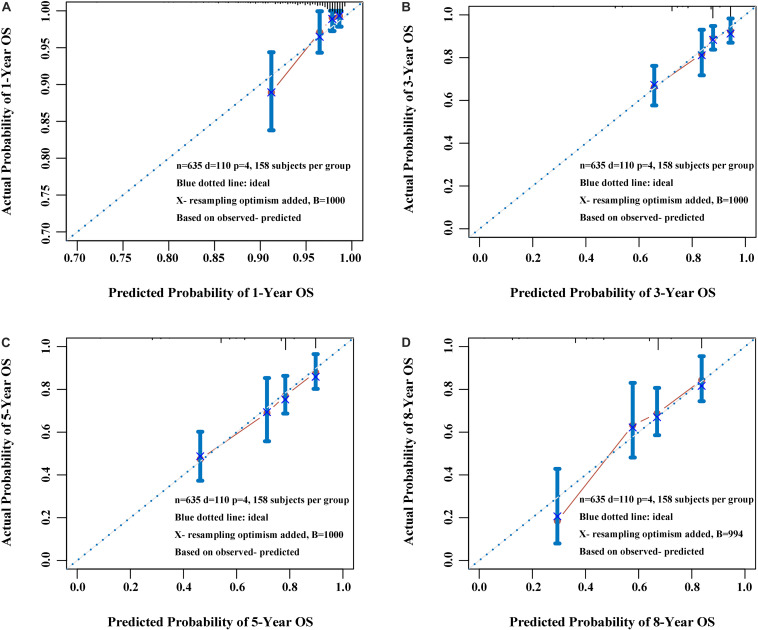
Calibration plot of the nomogram at 1 year **(A)**, 3 years **(B)**, 5 years **(C)**, and 8 years **(D)**. The calibration curves were well matched with the idealized 45° line.

**FIGURE 8:**
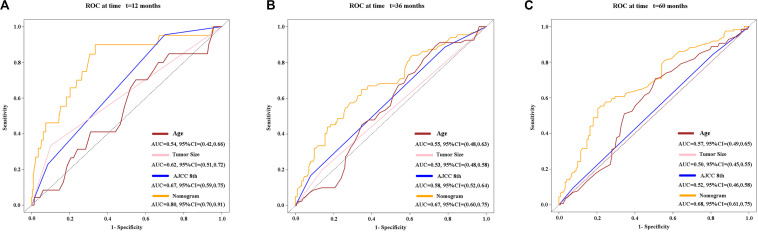
Time-dependent receiver operating characteristic (timeROC) curves at 1 **(A)**, 3 **(B)**, and 5 **(C)** years of overall survival based on the nomogram, age, tumor size, and AJCC 8th staging system. AJCC, American Joint Committee on Cancer.

Three variables (APS, tumor size, age), which were independent prognostic factors of OS, were integrated to construct a novel nomogram for predicting prognosis (Figure 6). The C-index for the nomogram for assessment of OS after ablation was 0.72 (95% CI 0.66, 0.77). The calibration plots for probability of survival at 1, 3, 5, and 8 years with 1000 cycles of bootstrapping were well matched with the idealized 45° line (Figure 7). Besides, we calculated individualized scores of each patient, which was the total score for those three prognostic variables. Time-dependent ROC curves at different times of OS, the AUC and C-index values, and the corresponding *P-*value suggested that the novel inflammation-based nomogram system improved the performance and discrimination in predicting the short-term or long-term prognosis of HCC patients within the Milan Criteria after curative ablation (Figures 3, 4C). Besides, the time-dependent ROC curves also showed that compared with age, tumor size, and the American Joint Committee on Cancer (AJCC) 8th staging system (3), the novel inflammation-based nomogram system has obvious advantages in predicting prognosis of HCC patients within the Milan Criteria after curative ablation at 1 year [compared with age (*P* < 0.001), tumor size (*P* = 0.004), and AJCC 8th staging system (*P* = 0.031)], 3 years [compared with age (*P* = 0.004), tumor size (*P* < 0.001), and AJCC 8th staging system (*P* = 0.028)], and 5 years [compared with age (*P* = 0.010), tumor size (*P* < 0.001), and AJCC 8th staging system (*P* < 0.001)] of OS (Figure 8).

## Discussion

In this study, we firstly found a novel inflammation-based score system—Albumin-Platelet Score (APS)—that has a significant advantage over others in predicting the long-term prognosis by systematically analyzing the pre-treatment clinical characteristics and the inflammatory indicators included in these inflammation-based scores (i.e., NLR, PLR, PNI, mGPS, GPS, PI, and CAR). Also, the nomogram based on the APS further improved the performance of predicting the prognosis of HCC patients within the Milan Criteria after ablation.

As we all know, inflammation promotes bad prognosis of HCC through induction of thrombocytopenia, lymphopenia, and resistance to chemotherapy (17–19). Therefore, inflammation-based prediction systems have great potential in predicting the prognosis of HCC patients (7–13). Especially in China, most cases of HCC are caused by potentially chronic HBV infection. However, few studies based on pretreatment inflammation-based markers focused on assessing the prognosis of HCC patients within the Milan Criteria after curative ablation. To bridge the gap, we systematically analyzed the pre-treatment clinical characteristics to find a significant pre-treatment inflammation-based markers to choose a more ideal treatment for HCC patients within the Milan Criteria.

The APS was an integrated indicator based on peripheral ALB level and PLT counts. In this study, reduced ALB level (ALB ≤ 37.7 g/L) and PLT counts (PLT ≤ 80 × 10^9^/L) were independent predictors of OS in HCC patients within the Milan Criteria after curative ablation. Platelets were involved in the pathogenesis of chronic liver disease through hemostasis and inflammatory processes. Kondo et al. (20) reported an important outcome of the accumulation of platelets in the liver with chronic hepatitis causing thrombocytopenia and liver fibrosis through the activation of hepatic stellate cells (HSCs). Therefore, thrombocytopenia was considered as an important feature of chronic liver disease and cirrhosis. In addition, thrombocytopenia was associated closely with the development of hepatocarcinogenesis (21). Furthermore, some studies suggested that thrombocytopenia was regarded as an inexpensive, valuable predictor for the recurrence, and survival in patients with HCC (22, 23). ALB was an important component of various liver function evaluation indicators, such as Child–Turcotte–Pugh classification, ALBI grade, and some inflammation-based score systems, such as GPS, mGPS, and PNI, and they were closely related to the prognosis of HCC (9, 10, 12, 14, 24). Therefore, the APS was an important predictive indicator of the efficacy of HCC undergoing ablation theoretically and practically. Besides, we conducted a correlation analysis between patient characteristics and the APS, and found that among HCC patients within the Milan Criteria after curative ablation with a higher APS, more patients had reduced WBC, neutrophil, and lymphocyte counts; increased CRP level; increased PNI, mGPS, GPS, and CAR; increased ALBI grade; cirrhosis; and increased AST and TBIL, suggesting a higher APS often with poorer immune response, an elevated inflammation status, and worse liver functional reserve. We also found that patients with cirrhosis have significantly reduced WBC, neutrophil, and lymphocyte counts than patients without cirrhosis, which suggested that leukopenia, neutropenia, and lymphopenia were also considered as important features of chronic liver disease and cirrhosis in HCC patients within the Milan Criteria, similar to the thrombocytopenia. In fact, some studies showed the HBV-encoded regulatory HBX protein was able to transactivate the IL-8 promoter, which promoted the IL-8 expression and elicited granulocytes, NK cells, and T-cell chemotaxis at the inflammatory regions, contributing to the development of liver damage (25–28). Therefore, we assumed that the accumulation of neutrophil in the liver with chronic hepatitis was also one of the important causes of neutropenia in early-stage HCC.

Based on the significance of APS, we established an easy-to-use nomogram based on three pretreatment clinical variables, including APS level, tumor size, and age, to assess the prognosis of HCC patients within the Milan Criteria after curative ablation. We also found that the novel inflammation-based nomogram system significantly improved the performance and discrimination in predicting the short-term or long-term prognosis of HCC patients within the Milan Criteria after curative ablation. Also, the nomogram system has more obvious advantages than AJCC 8th staging system in predicting OS of HCC patients within the Milan Criteria after curative ablation.

Besides, there are some considerations to consider when constructing the nomogram. To reduce the expected error in the predicted probability below 10%, the numbers of survival and death should be greater than 10 times the numbers of variables constructing the nomogram (29). The number of deaths was 110, which was more than 36.7 times the number of variables in our study. Considering the insufficient number of cases in the external validation group, we applied the internal validation with 1000 sets of bootstrap samples and its calibration curve and well verified the nomogram. Therefore, the nomogram system can help clinicians make good decisions, improve patient–physician communication, and even choose suitable HCC patients for clinical trials.

Although our findings were significant, there were several limitations to our study. First, the study was a retrospective study mainly based on HBV-infected population, so whether the APS could also predict the prognosis well in non-HBV-predominated HCC patients within the Milan Criteria after curative ablation is a question worthy of further verification. Second, the number of patients with mGPS 3 level, GPS 3 level, and PI 3 level were quite less, which may weaken the ability of mGPS, GPS, and PI in predicting the prognosis. Third, although the ideal cut-off values for these pre-treatment baseline variables were based on survival ROC, which could fit Cox proportional-hazards modeling to the status and the time of survival, this was a single-center study. Therefore, the prospective and multicentric external verification will be conducted to further verify this novel inflammation-based score—APS—and the nomogram based on the APS.

## Conclusion

This study is the first to find the novel inflammation-based score—APS—that was a better inflammation-based prognostic system than others (i.e., NLR, PLR, PNI, mGPS, GPS, PI, and CAR). Also, the nomogram based on the APS improved the performance of predicting the prognosis of HCC patients within the Milan Criteria after ablation.

## Data Availability Statement

The raw data supporting the conclusions of this article will be made available by the authors, without undue reservation.

## Ethics Statement

The studies involving human participants were reviewed and approved by the research ethics committee of the Sun Yat-sen University Cancer Center (SYSUCC). Written informed consent for participation was not required for this study in accordance with the national legislation and the institutional requirements.

## Author Contributions

SC and WF conceived and designed this study. WF provided the study material and access to patients. SC, WM, FC, LS, HQ, LX, and YW acquired the data. SC, WM, FC, LS, HQ, LX, YW, and WF analyzed the data and drafted the manuscript. All authors contributed to the draft and critically reviewed or revised the manuscript.

## Conflict of Interest

The authors declare that the research was conducted in the absence of any commercial or financial relationships that could be construed as a potential conflict of interest.

## Footnotes

1 https://www.r-project.org/

## Abbreviations

AFP: alpha-fetoprotein
ALB: albumin
ALBI: albumin-bilirubin
ALT: alanine aminotransferase
APS: Albumin-Platelet Score
AST: aspartate transaminase
AUC: area under the curve
CAR: C-reactive protein/albumin ratio
C-index: Harrell’s concordance index
CRP: C-reactive protein
GPS: Glasgow Prognostic Score
HBV: hepatitis B virus
HCC: hepatocellular carcinoma
mGPS: modified Glasgow Prognostic Score
MWA: microwave ablation
NLR: neutrophil to lymphocyte ratio
OS: overall survival
PI: Prognostic Index
PLR: platelet to lymphocyte ratio
PLT: platelet
PNI: Prognostic Nutritional Index
PT: prothrombin time
RFA: radiofrequency ablation
ROC: receiver operating characteristic
TACE: transarterial chemoembolization
TBIL: total bilirubin
WBC: white blood cell.
